# Traumatic tension pneumocephalus: a case report and perspective from Indonesia

**DOI:** 10.3389/fneur.2024.1339521

**Published:** 2024-02-07

**Authors:** Alphadenti Harlyjoy, Michael Nathaniel, Aryandhito Widhi Nugroho, Kevin Gunawan

**Affiliations:** ^1^Department of Neurosurgery, University Indonesia Hospital, Depok, West Java, Indonesia; ^2^Department of Surgery, Khairun University, Ternate, North Maluku, Indonesia

**Keywords:** tension pneumocephalus, traumatic brain injury, LMIC, Indonesia, global neurosurgery

## Abstract

Traumatic tension pneumocephalus is a rare and life-threatening complication of traumatic brain injury necessitating prompt diagnosis and neurosurgical treatment. Nevertheless, various possibilities for impedance in timely management, including patient-related barriers are commonly experienced in low-and middle-income countries setting. Here we presented a delay of management in traumatic tension pneumocephalus case due to initial refusal for emergency surgery. A 59-year-old male presented to the emergency department following a motorcycle accident fully alert with no neurological deficit. He acknowledged clear nasal discharge within 1 h after the initial trauma, but no rhinorrhea or otorrhea was present during physical examination. Head CT revealed extensive pneumocephalus with “Mount Fuji sign,” anterior skull base fracture, and frontal sinus fracture. The patient initially refused immediate surgical intervention due to excellent clinical condition and financial scare. Acute decrease of consciousness occurred 40 h post-trauma: GCS of 6 with slight dilatation of both pupils (4 mm) and sluggish pupillary reflex. Emergency bifrontal craniotomy, subdural air drainage, and dura mater tear repair were performed afterwards. Postoperative care was uneventful, with rapid improvement of consciousness and follow-up head CT showing minimal subdural fluid collection and absence of remaining pneumocephalus. The patient was discharged from the hospital after 7 days with GCS of 15 and GOS of 5, proving the importance of overcoming barriers for delay in delivering neurotrauma care in low-and middle-income countries.

## Introduction

Pneumocephalus is defined as pathological air collection in the cranial cavity. The air accumulation can reside in the epidural, subdural, subarachnoid, intraparenchymal, or intraventricular spaces (1–5). Etiological factors comprised of head injury, intracranial infection, and craniofacial operative procedures, including but not limited to neurosurgical and otorhinolaryngology interventions (3, 6). A report of 295 cases shows that the most common cause for pneumocephalus is trauma, accounting for 75% of the study population (7). Traumatic pneumocephalus is considered an uncommon pathology in traumatic brain injury (TBI) group, having its incidence contributing only to 0.5–9.7% of all TBI cases, and is primarily self-limiting (3, 4, 8). Nevertheless, complication could occur when the intracranial air collection generates significant increase in intracranial pressure (ICP), causing subsequent neurological deterioration (3, 4, 9). This emergency is identified as traumatic tension pneumocephalus, a serious complication that could lead to progressive brain compression, brain oxygen supply reduction, and brain herniation (6). Traumatic tension pneumocephalus requires urgent diagnosis and intervention, with predominantly good outcome if managed timely (8). Diagnostic and treatment delays will bring about poor neurologic outcomes and mortality (1, 9).

Immediate access to medical service for TBI is one of the unmet global neurosurgical necessities, with even greater delays in low- and middle-income countries (LMICs) due to social, cultural, financial, infrastructure, and disparity of resources issues (10, 11). According to the Global Burden of Disease study in 2017, trauma causes 4.5 million deaths and 759 per 100,000 individuals living with disability worldwide, with majority of those residing in LMICs (12, 13). It is estimated that the global incidence of TBI reached 69 million each year, and the South East Asia region is regarded as its largest contributor with the annual incidence of 18.3 million cases (14). This report examines a traumatic tension pneumocephalus case from Indonesia, the highest populated LMIC in South East Asia, providing further insight on the issues encountered during management of a rare TBI presentation in a developing country.

## Case

A 59-year-old male was admitted to a type 1 emergency department of a secondary-care hospital two hours after a motorcycle accident. He was admitted straight from the accident site by private-owned vehicle of a passerby, fully alert (Glasgow Coma Scale [GCS] of 15) without any neurological deficit and motor score of 5 in all extremities. The patient reported a history of rhinorrhea in the first hour after the accident, but no cerebrospinal fluid was visible in the nasal cavity or auditory canal. Vital signs, other physical examinations, and laboratory results were within normal range.

**Figure 1:**
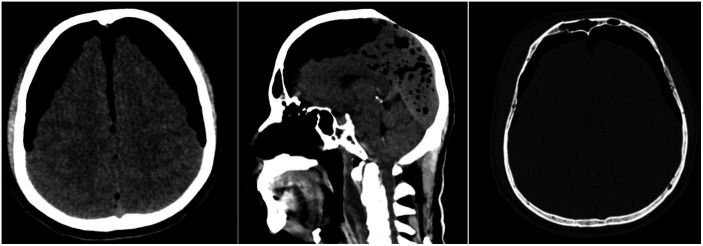
Emergency CT scan showed tension pneumocephalus with “Mount Fuji sign,” diffuse air bubbles, anterior skull base fracture, and frontal sinus fracture.

Computerized tomography (CT) imaging of the head revealed extensive pneumocephalus with “Mount Fuji sign,” together with fractures of the anterior skull base and frontal sinus anterior–posterior wall. Smaller diffuse bubbles were also present throughout the posterior part of the interhemispheric space (Figure 1).

The on-call neurosurgeon assessed the case as a tension pneumocephalus indicated for emergency craniotomy. Patient initially refused neurosurgical intervention and hospital care due to excellent clinical condition and financial scare, but eventually agreed to be admitted for a 48-h post-trauma monitoring. Clinical deterioration occurred 40 h after trauma. He became lethargic and had widened pulse pressure, which in the next 30 min progressed into unresponsiveness (GCS of 6), slight dilatation of both pupils (4 mm), and sluggish pupillary reflex.

Emergency bifrontal craniotomy was carried out immediately. Initial intraoperative findings were subdural air collection and dura mater tear in the anterior skull base. Consequent subdural air drainage and repair of the dura mater were performed.

**Figure 2:**
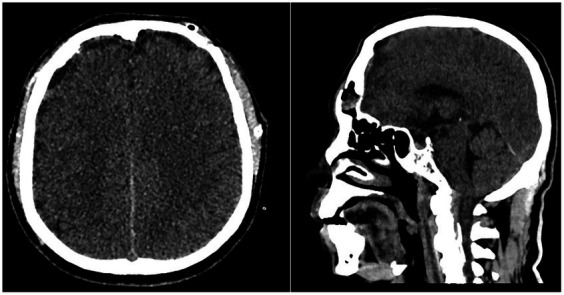
The follow-up head CT scan showed thin subdural collection and minimal remains of pneumocephalus.

**Figure 3:**
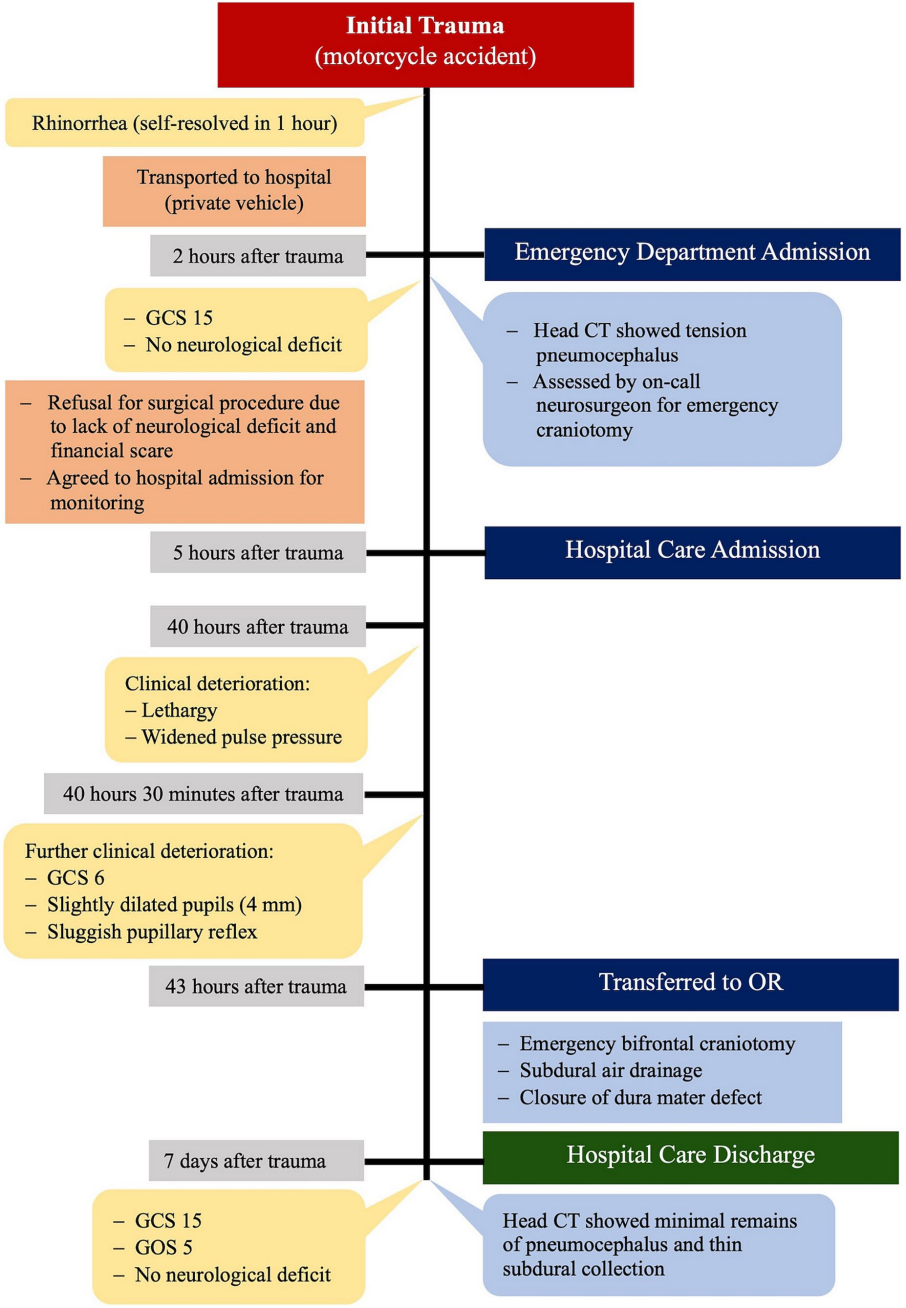
Timeline of the case.

The patient regained consciousness rapidly and was fully alert the day after surgery. He had an uneventful recovery and was discharged from the hospital after 7 days, with discharge GCS of 15 and Glasgow Outcome Score (GOS) of 5. A follow-up head CT scan showed thin subdural collection and minimal remains of pneumocephalus in the frontal region (Figure 2). Timeline of the case is illustrated in Figure 3.

## Discussion

Efforts to deliver optimal treatment for neurotrauma cases in LMICs are often met with a myriad of challenges, including patient-related barriers (10). This report presents a delay of definitive treatment for traumatic tension pneumocephalus encountered in a hospital with type 1 emergency care due to patient’s initial reluctance to undergo immediate neurosurgical intervention. The main pathology in this case is identified at an early stage through proper imaging modalities available at the hospital. Pneumocephalus is most easily diagnosed through a head CT scan, where air appears darker than CSF, with attenuation values of-1000 Hounsfield units (8, 9, 15). If the air resides in subdural space, extends downward to the interhemispheric fissure, and separates both frontal lobes anteriorly, it will show a unique appearance referred to as the “Mount Fuji sign.” This bilateral compression of the frontal lobe had been associated with tension pneumocephalus (1, 2, 8, 9). The case depicted in this report exhibited the pathognomonic “Mount Fuji sign” on head CT scan, making tension pneumocephalus a definite diagnosis.

Identification of underlying disease at earlier stage significantly aids correct planning for patient’s management. Patients with untreated tension pneumocephalus are at great risk for further complications due to progressive brain compression (2, 5, 6, 16). Spectrum of neurological deterioration ranges from altered mental status and lethargy (from reduced oxygen supply), stupor and coma (from brain herniation), to death of the patient (2, 4, 6, 8, 16). In delayed or slow-progressing cases, it is important to recognize early signs of increased ICP, including headache, nausea, and vomiting (8, 16). More significant changes such as agitation, delirium, decreased vision, pupillary changes, or evidence of Cushing reflex should be noted as well (8, 9, 16). In his initial state, the patient presented in this report did not exhibit any neurological deficits, which contributes to his refusal for emergency surgical intervention despite being given thorough explanation by the on call neurosurgeon. Lack of comprehension regarding course of the disease and importance of treatment, paired with patient’s financial instability, are common socioeconomic issues causing hesitance to receive medical care in LMICs (11, 17). Considering the imminent life threat identified through head CT, the neurosurgeon persuaded the patient to agree to a 48-h hospital admission for monitoring. As anticipated, the patient then experienced the predicted clinical deteriorations due to ICP raise in the form of lethargy and widened pulse pressure, which rapidly progressed into loss of consciousness and slightly dilated pupils. The 40-h delayed neurological decline provides the patient’s family the conviction required to finally opt for surgery. Fortunately, urging for hospitalization, instead of allowing the patient to be discharged from medical care earlier on, saves significant amount of preparation time required for emergency neurosurgical procedure.

Urgent surgical decompression is mandatory for tension pneumocephalus (1, 2, 5, 6). Various surgical options are available, including craniotomy, burr hole, endoscopy, and ventriculostomy (2, 5, 9, 16). Irrespective of the procedure, emergent evacuation of compressive intracranial air is crucial for optimal outcome (5, 6). If possible, identification and repair of the dura mater defect is considered as definitive treatment that should be performed as well, and could also be achieved through endoscopic endonasal procedure (1, 9, 16, 18, 19). In this patient’s case, bifrontal craniotomy, subdural air drainage, and definitive closure of the dura mater defect, were the chosen course of action. Despite considerable hours of delay, the procedure proved to be effective, enabling the patient to be discharged from hospital care 7 days after initial trauma with GCS of 15 and GOS of 5.

This case report shows the significance of patient-related issues in LMICs. Despite attaining the global neurotrauma proposed standard of 4-h window and Lancet Commission of Global Surgery recommendation of 2 hours access to care, added with availability of sufficient diagnostic measures, hospital facilities, and neurosurgeon, the patient still experienced a 40-h delay due to initial refusal for surgery (20, 21). It is important to note that previous accounts of traumatic tension pneumocephalus cases had described the outcome of mortality in absence of neurosurgical intervention (1–4, 6).

Delays in providing best neurotrauma care in LMICs is highly influenced by patient-related barriers (10, 11, 17). Presence of stigma and traditional beliefs concerning disease processes, absence of health education and social support, together with possibility of patient’s financial detriments due to seeking care, are few of the main problems encountered in daily medical practice of developing countries, including Indonesia (11, 17, 22). Limited understanding of medical problems where certain perspective that the disease contracted does not have significant negative impact on patients’ lives results in reluctance for receiving medical care (23). The fact that current demography of LMICs TBI patients are dominated by breadwinners of the family consequently adds additional hindrance, since hospitalization would result in income regression and unemployment (12, 13, 17, 24). A survey involving Indonesian neurosurgeons found that 27.3% of its participants regarded TBI as the worst cause for economic hardship among patients. Even though public neurosurgical centers in Indonesia are mandated by legislation to provide healthcare at minimum or zero cost, seeking healthcare would put the poverty-ridden population under serious financial burden due to transportation costs and caregiving expenses (17).

Being the world’s largest archipelagic country, geography-related issues become the most notable reason for delay in reaching care in Indonesia, which expands through 1,892,410.09 km^2^, 17,001 islands, and 37 provinces, with a population of 275,773,000 people. Population density differs highly, where 56% of the citizens live on Java Island (geographically occupies only 7% of Indonesia’s territory), while the whole eastern part of Indonesia (26% of the country’s area, consisting of 4 provinces, Maluku, North Maluku, Papua, and West Papua) only composes 3.2% of Indonesia’s population (25). That discrepancy, coupled with suboptimal decentralized government policies, causes the lack of support for prehospital transport in rural regions. Studies conducted in Bandung [urban city of West Java province, population density 1,334 people/km (2)] and Belitung (rural city of Bangka Belitung province, population density 90 people/km^2^) would best illustrate the difference by having 83.8 and 10.4%, respectively, of their TBI patients admitted to the hospital by ambulance (17, 26, 27).

Shortage of neurosurgical expertise and suboptimal hospital service also contributes significant barriers to the system. Neurosurgeon-to-population ratio is 1:725,000 (371 neurosurgeons), very low compared to the recommended 1:100,000, with the rural community in a greater disadvantage due to its uneven distribution (28). Over 257 (69.2%) neurosurgeons practices in Java, with most hospitals offering CT scan service, whereas Papua only has five CT scan machines and four neurosurgeons (8, 29). North Maluku only has one neurosurgeon and one CT scan machine, of which is located 200 kilometers away from the neurosurgical center. Patients would need to undergo 5 hours of road travel and thirty minutes of sea fare each way, violating the global recommendations for safe neurosurgical evaluation and care (20, 21).

The issues mentioned above should be the topic of discussion between local government and medical professionals involved in Indonesia’s neurosurgical care. TBI accounts for 11.9% of trauma cases in Indonesia, making it the third most common cause of traumatic injury for the nation (30). Local 1-year single-centered studies from the national referral hospital and three other secondary-care hospitals in Belitung, Bali, and Papua presented 157, 270, 525, and 393 cases, respectively (26, 31–33). Mortality rate ranges from 7.6 to 29.23%, where the lowest percentage is found in the national referral hospital, and the highest occurred in a secondary-care hospital in Bandung (26, 27, 31, 32, 34–36). Higher patient volume and mortality rate shown in secondary-care hospitals revealed the underperformance of overwhelmed medical service, further proving the need for upgrades in the health care system.

Medical professionals’ better insight into difference in Indonesian native’s characters and concerns would produce more effective methods in obtaining patients’ consent and compliance for comprehensive treatment of their diseases. For the patient in this report, emphasis on major possibility for mortality and perspective concerning possible impoverishing expenditure (e.g.: death of the patient would put his family under a more serious financial loss compared to taking a leave from work and opting for surgery now) should have been the primary focus of doctor-patient discussion, mitigating the delay in administering optimal neurotrauma care.

Universal improvement for the nation’s neurotrauma service should start with comprehensive appraisal of all possible issues, relating to patient, infrastructure, physician, and health resources for every province of Indonesia. The insights acquired from the framework should then be utilized by the stakeholders in creating tangible programs tailored to each region’s needs.

## Conclusion

Traumatic tension pneumocephalus is a rare presentation of TBI that requires timely diagnosis and treatment. Efforts to reduce delays of TBI management in LMICs should incorporate thorough evaluation for barriers relating to patient, infrastructure, physician, and health resources countrywide.

## Data availability statement

The original contributions presented in the study are included in the article/supplementary material, further inquiries can be directed to the corresponding author.

## Ethics statement

Written informed consent was obtained from the individual(s) for the publication of any potentially identifiable images or data included in this article.

## Author contributions

AH: Writing – original draft. Writing – review & editing. MN: Writing – review & editing. AN: Writing – original draft. KG: Writing – original draft.
